# Prevalence of Dysmenorrhea and Its Association With Dietary Habits Among Female Students of Mid‐West University, Surkhet, Nepal: An Analytical Cross‐Sectional Study

**DOI:** 10.1002/hsr2.72486

**Published:** 2026-05-04

**Authors:** Rashmi Pant, Mamta Verma, Rima Mishra, Ganesh Bhandari

**Affiliations:** ^1^ Pokhara University, Kaski Pokhara Nepal; ^2^ Tapei Medical University Taipei City Taiwan

**Keywords:** dietary habits, dysmenorrhea, menstrual health, menstrual pain, university students

## Abstract

**Background and Aims:**

Dysmenorrhea is a common gynecological condition affecting young women and is associated with reduced academic performance and quality of life. This study aimed to determine the prevalence of dysmenorrhea and examine its association with dietary habits among female students at Mid‐West University, Surkhet, Nepal.

**Methods:**

An analytical cross‐sectional design conducted from 29 October 2023 to 30 November, 2023, and data were collected from 207 menstruating bachelor‐level students using an interviewer‐administered questionnaire and standard anthropometric measurements. Pain severity was assessed using a Visual Analog Scale.

**Results:**

The prevalence of dysmenorrhea was 88.4% (183/207), and nearly half of affected students (43.7%) experienced severe menstrual pain. Family history of dysmenorrhea demonstrated a significant association with pain severity (*p* = 0.004), suggesting hereditary influence, whereas menstrual characteristics including age at menarche, cycle regularity, and bleeding duration were not associated with pain intensity. Dietary practices, including meal frequency, food avoidance during menstruation, frequency of consumption of major food groups, and nutritional status based on Body Mass Index, did not show significant associations with dysmenorrhea severity. Self‐care practices such as sleep (26.3%), heat therapy (24.8%), and warm beverages (15.2%) were the most commonly used pain‐relief methods, while medical consultation remained low (19.7%).

**Conclusion:**

The findings highlight dysmenorrhea as a substantial health concern among university students and indicate that biological and familial factors may play a more influential role in pain severity than dietary behaviors. Strengthening menstrual health education, improving access to evidence‐based pain management, and integrating menstrual health support within university services are essential to reduce the academic and psychosocial burden of dysmenorrhea among young women in Nepal.

## 1 Introduction

Dysmenorrhea, defined as painful menstruation of uterine origin is one of the most prevalent gynecological conditions affecting adolescents and young women worldwide, representing a substantial yet often overlooked public health concern. Global estimates indicate that 70% to 91% of women of reproductive age experience menstrual pain, with up to 45% reporting moderate to severe symptoms that impair daily functioning, academic participation, and quality of life [1, 2, 3]. Recent meta‐analyses show that dysmenorrhea accounts for significant school absenteeism, reduced productivity, and psychosocial distress, making it a leading cause of disability among adolescent girls globally [4, 5]. The burden is particularly pronounced in low‐ and middle‐income countries (LMICs), where limited access to menstrual health services and sociocultural stigma surrounding menstruation exacerbate unmet needs for adequate care [6].

Physiologically, primary dysmenorrhea results from excessive endometrial prostaglandin production, which induces uterine hypercontractility, ischemia, and pain [7]. Secondary dysmenorrhea is associated with underlying pathologies such as endometriosis, pelvic inflammatory disease, or fibroids [8]. Evidence shows that the severity and frequency of dysmenorrhea are influenced by multiple factors including age, early menarche, stress, physical inactivity, parity, and lifestyle behaviors [9]. Dietary habits have emerged as an important modifiable determinant, with growing research examining the role of nutrient intake, meal patterns, and food types in modulating inflammatory pathways related to menstrual pain [10, 11, 12].

Previous literature suggests that diets high in fruits, vegetables, omega‐3 fatty acids, and micronutrients such as magnesium, vitamin D, and calcium are associated with reduced dysmenorrhea severity, while frequent consumption of fast foods, sugary snacks, caffeine, and high‐fat diets is linked with increased risk and intensity of menstrual pain [10, 13, 14, 15]. Although evidence is mixed, several observational and interventional studies provide biological plausibility through mechanisms involving inflammation regulation, hormonal balance, and prostaglandin synthesis [13, 16]. Despite growing interest, findings remain inconsistent across populations due to methodological differences, varying dietary habits, and limited research among university‐aged women in LMICs.

In South Asia, dysmenorrhea is highly prevalent yet under‐researched, particularly in rural and semi‐urban areas where menstrual health awareness and healthcare access are limited. Studies from India, Pakistan, and Bangladesh report prevalence rates ranging from 70% to 90%, with significant proportions of students reporting disruptions to academic performance and daily activities [17, 18, 19]. In Nepal, dysmenorrhea is similarly common, with studies showing prevalence between 72% and 83% among adolescents and university students [20, 21, 22].

However, most available research in Nepal has predominantly focused on prevalence and associated factors among school and university students in urban or institutional settings, with limited attention to dietary behaviors as potential contributors to menstrual pain [23, 24].

The Karnali region, where Mid‐West University is located, faces unique nutritional challenges including limited dietary diversity, food insecurity, and restricted access to fresh fruits and vegetables [25, 26, 27]. Given these contextual factors, understanding the relationship between dietary patterns and dysmenorrhea in this setting is crucial. Evidence specific to university students in Surkhet is scarce, creating a gap in knowledge that limits context‐specific recommendations for menstrual health programs.

This study aims to estimate the prevalence of dysmenorrhea among female students at Mid‐West University, Surkhet, and to examine its association with dietary habits. Generating locally relevant evidence is essential to inform campus‐based health initiatives, nutritional counselling programs, and broader public‐health strategies targeting menstrual well‐being among young women in Nepal.

## 2 Methodology

### 2.1 Study Design and Study Setting

An analytical cross‐sectional study design was employed to assess the prevalence of dysmenorrhea and its association with dietary habits among female students of Mid‐West University, Surkhet, Nepal. This design was chosen to obtain a snapshot of menstrual characteristics, pain experiences, and dietary patterns within the study population. The study was carried out from 29 October, 2023 to 30 November, 2023.

The study took place at university's main campus in Birendranagar, which serves a diverse student population drawn from rural, peri‐urban and remote mountainous areas of Karnali Province. This diversity, along with the region's distinct socio‐geographical context, provides an appropriate setting for examining menstrual health and nutrition‐related patterns among young women.

### 2.2 Study Population and Eligibility Criteria

The study included female bachelor‐level students aged 18–30 years who had attained menarche and experienced menstruation within the last 3 months. Only students who were present during the data collection period and provided informed written consent were included.

Students were excluded if they were pregnant at the time of the study, had a known diagnosis of chronic gynecological conditions such as endometriosis, polycystic ovarian syndrome (PCOS), pelvic inflammatory disease, or uterine fibroids, or were currently under treatment for secondary dysmenorrhea. Additionally, students who were severely ill, unable to participate in the interview, or unwilling to provide complete information were excluded.

### 2.3 Sample Size and Sampling Procedure

The sample size was calculated using the single population proportion formula, assuming a dysmenorrhea prevalence of 84% from a previous Nepal‐based study [21], with a 95% confidence interval and a 5% margin of error. The final required sample was 207, which was achieved during data collection.

A convenience sampling approach was used. Female students who were available during the data collection period and met the eligibility criteria were approached and recruited for participation.

### 2.4 Data Collection Tools and Procedures

Data were collected using a structured interviewer‐administered questionnaire, which covered socio‐demographic characteristics, menstrual history, the presence and severity of dysmenorrhea, pain management strategies, dietary practices, and nutritional status.

Pain intensity was measured using a standard Visual Analog Scale (VAS), a validated, widely used unidimensional tool for assessing subjective pain. The VAS consisted of a horizontal 10‐cm (100 mm) line anchored at the left end by “no pain” (0 mm) and at the right end by “worst pain imaginable” (100 mm). Participants were instructed to mark a point on the line that best represented their typical menstrual pain level over the past 3 months, based on recall of their most recent menstruation(s). The distance from the left anchor to the participant's mark was measured in millimetres using a standard ruler and recorded as the pain score.

Based on standard cut‐points used in dysmenorrhea research, pain severity was categorized as: mild (0–30 mm), moderate (31–60 mm), and severe (61–100 mm). The VAS has demonstrated good test‐retest reliability (intraclass correlation coefficient > 0.80) and convergent validity with other pain scales in menstrual pain research.

### 2.5 Questionnaire Development

The structured questionnaire was developed through a multi‐step process. First, a comprehensive literature review was conducted to identify relevant domains and previously validated questions on dysmenorrhea, menstrual characteristics, dietary habits, and pain management [10, 21]. Second, the initial draft questionnaire was organized into five sections: (A) sociodemographic characteristics, (B) menstrual history and dysmenorrhea assessment, (C) pain severity using the Visual Analog Scale (VAS), (D) pain management strategies, and (E) dietary practices including meal patterns, food frequency, and food avoidance during menstruation.

Third, content validity was assessed by a panel of three experts: one public health researcher with expertise in reproductive health, one nutritionist, and one experienced academic in the Department of Home Science at Mid‐West University. Based on their feedback, ambiguous items were reworded, redundant questions were removed, and the order of sections was rearranged to improve logical flow. Fourth, the questionnaire was pre‐tested on 20 female students (approximately 10% of the target sample size) from a similar population at a different faculty not included in the main study. Pre‐testing assessed clarity, comprehension, completion time (average 20–25 min), and face validity. Minor modifications were made to wording and response options based on pre‐test feedback. The final questionnaire was prepared in English, as all participants were proficient in English due to university‐level instruction.

### 2.6 Study Variables

The primary outcome variable was dysmenorrhea, assessed based on its presence and severity using VAS scores. Independent variables included socio‐demographic factors (age, ethnicity, religion, family type, family size, faculty), menstrual characteristics (age at menarche, cycle regularity, cycle length, duration of bleeding, family history of dysmenorrhea), dietary habits (meal frequency, meal skipping, foods consumed and avoided during menstruation), and anthropometric indicators (BMI). Dysmenorrhea prevalence in this study refers to period prevalence over the 3 months preceding data collection. Participants were classified as having dysmenorrhea if they reported experiencing menstrual pain during any of their last three menstrual cycles. This 3‐month recall period was chosen to balance memory accuracy against the need to capture typical pain patterns while minimizing recall bias.

### 2.7 Assessment of Nutritional Status

Nutritional status was evaluated using BMI, calculated as weight in kilograms divided by height in meters squared. WHO BMI classification was used to categorize participants into underweight, normal, overweight, and obese groups.

### 2.8 Data Management and Statistical Analysis

Collected data were coded and entered into SPSS version 26 for statistical analysis. Descriptive statistics including frequencies and percentages were used to summarize socio‐demographic profiles, menstrual patterns, pain characteristics, dietary patterns, and nutritional status. The Chi‐square test of independence was applied to assess associations between categorical variables, and statistical significance was determined at *p* < 0.05.

All continuous variables (age, age at menarche, bleeding duration, pain duration) were categorized into clinically meaningful groups prior to analysis, as shown in Tables 1, 2, 3. The chi‐square test of independence was therefore appropriate for all associations examined, as both the exposure and outcome variables were categorical. Fisher's exact test was used where any cell had an expected count less than 5.

**Table 1:** Sociodemographic characteristics of respondents (*N* = 207).

Variable	*n*	%
Age (years)		
18–21	139	67.1
22–25	45	21.7
26–30	23	11.1
Religion		
Hindu	192	92.8
Buddhist	6	2.9
Christian	9	4.3
Ethnicity		
Brahmin	55	26.6
Chhetri	83	40.1
Janajati	46	22.2
Dalit	23	11.1
Marital status		
Married	42	20.3
Unmarried	165	79.7
Family type		
Nuclear	101	48.8
Joint	102	49.3
Extended	4	1.9
Faculty		
Education	87	42.0
Humanities	78	37.7
Science & Technology	42	20.3

**Table 2:** Menstrual characteristics of respondents (=207).

Variable	*n*	%
Dysmenorrhea		
Yes	183	88.4
No	24	11.6
Family history		
Yes	96	46.4
No	111	53.6
Age at menarche (years)		
≤ 10	3	1.4
11–13	85	41.1
14–16	116	56.0
≥ 17	3	1.4
Cycle regularity		
Regular	170	82.1
Irregular	37	17.9
Cycle interval (days)		
< 28	15	6.8
28–30	149	72.0
> 30	6	2.9
Bleeding duration (days)		
2–3	91	44.0
4–5	95	45.9
6–7	21	10.1
Menstruation‐related symptoms^a^		
Nausea	12	2.0
Back pain	149	24.3
Vomiting	20	3.3
Cramps	64	10.4
Abdominal pain	93	15.2
Irritability	60	9.8
Headache	24	3.9
Mood swings	100	16.3
Diarrhea	7	1.1
Change in appetite	42	6.9
Bloating	42	6.9

^a^ Multiple responses permitted (each participant could report ≥ 1 symptom).

**Table 3:** Pain level and pain management characteristics among respondents (*n* = 182 respondents with dysmenohhrea).

Variable	*n*	%
Pain severity		
Mild	37	20.2
Moderate	66	36.1
Severe	80	43.7
Onset of pain		
1 day before	49	26.8
2 days before	25	13.7
3 days before	23	12.6
On day of menstruation	67	36.6
2nd day	17	9.3
3rd day+	2	1.1
Duration of pain		
1–3 days	151	82.5
4–6 days	32	17.5
Pain management method^a^		
Sleep	109	26.3
Heat therapy	103	24.8
Warm beverage	63	15.2
Massage	45	10.8
Shower	32	7.7
Special food	32	7.7
Physical activity	13	3.1
Gadgets	10	2.4
None	8	1.9
Medicines used for pain relief^b^ (*n* = 26 medication users)		
Ibuprofen	4	15.4
Flexon (ibuprofen + paracetamol)	1	3.8
Mefenamic acid	19	73.1
Paracetamol	2	7.7
Sought medical care/consulted gynecologist		
Yes	36	19.7
No	147	80.3

^a^ Multiple responses permitted (each participant could report ≥ 1 pain management method).

^b^ Among those who reported using medication.

### 2.9 Ethical Considerations

Ethical approval for the study was obtained from the Department of Home Science, Mid‐West University (Ref 52‐081/82). Participants were informed about the purpose and procedures of the study, and written consent was obtained prior to data collection. Confidentiality, privacy, and anonymity were ensured throughout the study, and participation was entirely voluntary.

## 3 Results

### 3.1 Sociodemographic Characteristics

As shown in Table 1, A total of 225 female bachelor‐level students were approached during the data collection period. Of these, 18 students were excluded: 10 did not meet the eligibility criteria (8 had irregular cycles with no menstruation in the preceding 3 months; 2 had known diagnoses of PCOS), and 8 declined to participate (4 cited lack of time, 4 expressed discomforts discussing menstrual health). The remaining 207 students provided informed consent and completed the full interview, yielding a response rate of 92.0% (207/225). Non‐respondents did not differ significantly from respondents in terms of age or faculty distribution based on available demographic information. Most respondents were 18–21 years old (67.1%), followed by those aged 22–25 years (21.7%). The majority identified as Hindu (92.8%), and the largest ethnic group was Chhetri (40.1%), followed by Brahmin (26.6%) and Janajati (22.2%). Most participants were unmarried (79.7%), and family structure was almost evenly split between nuclear (48.8%) and joint families (49.3%). Respondents were enrolled across three faculties, with the Faculty of Education representing the largest proportion (42.0%), followed by Humanities (37.7%) and Science and Technology (20.3%).

### 3.2 Menstrual Characteristics

The menstrual characteristics of the respondents are presented in Table 2. The period prevalence of dysmenorrhea was seen in large majority of students (88.4%) reported experiencing menstrual pain, whereas only 11.6% did not experience dysmenorrhea. Nearly half of the participants (46.4%) also reported a family history of dysmenorrhea, indicating a notable hereditary pattern within the study population. More than half of the students experienced menarche between 14 and 16 years of age (56.0%), and the majority reported having regular menstrual cycles (82.1%). Most participants also described a typical cycle length of 28–30 days (72.0%) and a bleeding duration of 4–5 days (45.9%). A range of menstruation‐related symptoms was reported, with back pain being the most common (24.3%), followed by mood swings (16.3%), abdominal pain (15.2%), cramps (10.4%), irritability (9.8%), and bloating (6.9%).

### 3.3 Pain Level and Pain Management Characteristics

The distribution of pain intensity and pain management related characteristics among respondents with dysmenorrhea is shown in Table 3. Nearly half of the affected students experienced severe pain (43.7%), while 36.1% reported moderate pain and 20.2% reported mild pain. Pain most commonly began on the first day of menstruation (36.6%), followed by onset 1 day prior (26.8%). In terms of duration, the majority of participants experienced pain lasting 1–3 days (82.5%), indicating that short‐duration but often intense pain was typical within this population. Almost all participants reported using at least one method to relieve menstrual pain (95.6%). The most commonly adopted approaches were sleep (26.3%) and heat therapy (24.8%), followed by warm beverages (15.2%) and massage (10.8%). Among those who used medications, mefenamic acid was the predominant choice (73.1%). Notably, only 19.7% of respondents sought medical consultation for their menstrual pain, indicating a strong preference for self‐care practices within this population.

### 3.4 Dietary Habits, Food‐Frequency Consumption

The general dietary patterns of the respondents are presented in Table 4. The majority of students reported following a non‐vegetarian diet (75.8%), while just over one‐third adhered to a vegetarian diet. Most participants consumed three meals per day (62.8%), reflecting relatively consistent meal routines. Daily consumption of cereals was universal among all respondents (100%), and frequent intake of vegetables, fruits, dairy products, and processed foods was also commonly observed, indicating a varied dietary profile within the study population.

**Table 4:** Dietary habits (*n* = 207).

Variable	*n*	%
Food habit		
Vegetarian	50	24.2
Non‐vegetarian	157	75.8
Meals/day		
3 meals	130	62.8
4 meals	49	23.7
≥ 5 meals	28	13.5

### 3.5 Food‐Frequency Consumption

The food‐frequency distribution of major food groups is presented in Table 5. Cereals were consumed daily by all respondents, while the intake of legumes, fruits, vegetables, dairy products, and processed foods varied widely. Although vegetables and fruits were consumed frequently, the consumption of processed foods and sugary beverages remained notably high among a substantial proportion of students.

**Table 5:** Food‐frequency consumption (*n* = 207).

Food group	Daily	1–2×/week	3–4×/week	Never
Cereals & grains	207 (100%)	—	—	—
Legumes	32 (15.5%)	79 (38.2%)	81 (39.1%)	15 (7.2%)
Green leafy vegetables	83 (40.1%)	54 (26.1%)	65 (31.4%)	5 (2.4%)
Other vegetables	154 (74.4%)	16 (7.7%)	37 (17.9%)	0 (0%)
Meat & poultry	16 (7.7%)	76 (36.7%)	65 (31.4%)	50 (24.2%)
Dairy products	70 (33.8%)	79 (38.2%)	45 (21.7%)	13 (6.3%)
Fruits	63 (30.4%)	64 (30.9%)	76 (36.7%)	4 (1.9%)
Sugary beverages	14 (6.8%)	110 (53.1%)	47 (22.7%)	36 (17.4%)
Processed foods	41 (19.8%)	87 (42.0%)	74 (35.8%)	5 (2.4%)

### 3.6 Diet Practices During Menstruation and Nutritional Status

Dietary practices during menstruation are summarized in Table 6. More than half of the respondents reported consuming three meals per day (55.6%) during menstruation, indicating minimal disruption to regular meal patterns for most students. However, 20.3% avoided certain foods during this time, with spicy foods (35.7%) and sour foods (23.8%) being the most commonly restricted items. Only a small proportion of participants (11.6%) consumed specific foods to alleviate discomfort, most frequently hot soup (37.5%), followed by chocolate and other warm beverages. Overall, 12.1% of respondents reported skipping meals during their menstrual period. Among those who skipped meals, lunch was the most frequently omitted meal (56%), followed by breakfast (40%) and snacks (4%). The primary reasons for skipping meals included loss of appetite (38.5%), menstrual pain (26.9%), bloating (15.4%), and dizziness (15.4%), suggesting that physical discomfort played a central role in altering eating behaviors during menstruation. The distribution of respondents’ nutritional status based on BMI is shown in Table. The majority of students fell within the normal BMI range (73.4%), indicating generally adequate nutritional status among most participants. However, 18.4% were classified as underweight, while smaller proportions were categorized as overweight (6.3%) or obese (1.9%). These findings suggest that although most respondents had a normal BMI, a notable minority may be at nutritional risk.

**Table 6:** Dietary practices during menstruation and Nutritional Status (*n* = 207).

Variable	*n*	%
Avoid specific foods during menstruation		
Yes	42	20.3
No	165	79.7
Commonly avoided foods^a^ (*n* = 42)		
Spicy	15	35.7
Sour	10	23.8
Cold drinks	4	9.5
Eggs	3	7.1
Special foods eaten		
Yes	24	11.6
No	183	88.4
Commonly consumed special foods^a^ (*n* = 24)		
Hot soup	9	37.5
Dark chocolate	6	25.0
Chocolate	5	20.8
Skip meals		
Yes	25	12.1
No	182	87.9
Skipped meal (*n* = 25)		
Breakfast	10	40.0
Lunch	14	56.0
Snacks	1	4.0
Reason for skipping meal (*n* = 25)		
Appetite loss	10	38.5
Pain	7	26.9
Bloating	4	15.4
Dizziness	4	15.4
Nutritional status based on BMI		
Underweight (< 18.5)	38	18.4
Normal (18.5−24.9)	152	73.4
Overweight (25.0−29.9)	13	6.3
Obese (> 30.0)	4	1.9

*Note:* Association between pain severity and sociodemographic variables, menstrual characteristics and dietary habits, nutritional status and food frequency.

^a^ Multiple responses permitted.

Table 7 presents the relationship between pain severity and the respondents sociodemographic variables, menstrual characteristics and dietary habits, nutritional status and food frequency. None of the examined variables including age, religion, ethnicity, or marital status showed a significant association with pain severity. However, family type demonstrated a statistically significant relationship (*p* = 0.010), suggesting that household structure may influence menstrual pain experiences. On the other hand, variables such as age at menarche, menstrual regularity, bleeding duration, onset of pain, and duration of pain were not significantly associated with pain severity. In contrast, family history of dysmenorrhea showed a significant association (*p* = 0.004), indicating a possible hereditary component influencing pain intensity. None of the dietary factors including overall food habit, number of meals per day, meal skipping, or frequency of specific food groups were significantly associated with dysmenorrhea severity (all *p* > 0.05). Similarly, BMI showed no meaningful association with pain severity. These findings suggest that, within this study population, dietary behaviors and nutritional status did not play a measurable role in determining the intensity of dysmenorrhea.

**Table 7:** Association between pain severity and sociodemographic variables (*n* = 207).

Variable	Category	*p*‐value
Socio‐demographic characteristics		
Age	18–30 years	0.49
Religion	Hindu/Other	0.40
Ethnicity	Brahmin–Dalit	0.18
Family type	Nuclear/Joint/Extended	**0.01**
Marital status	Married/Unmarried	0.44
Menstrual Characteristics		
Age at menarche	≤ 10 to ≥ 17	0.28
Regularity	Regular/Irregular	0.18
Bleeding duration	2–7 days	0.30
Onset of pain	Before/At/After	0.70
Duration of pain	1–6 days	0.11
Family history	Yes/No	**0.004**
Dietary habits, nutritional status and food frequency		
Food habit		0.56
Meals per day		0.47
Meal skipping		0.07
BMI		0.30
Frequency of legumes		0.66
Green leafy vegetables		0.81
Other vegetables		0.89
Meat & poultry		0.95
Dairy products		0.17
Fruits		0.99
Sugary beverages		0.57
Processed foods		0.72

*Note:* All continuous variables were categorized prior to analysis; no continuous variables were entered directly into chi‐square tests. Bold values indicate statistically singificant at *p*‐value < 0.05.

## 4 Discussion

This study examined the prevalence of dysmenorrhea and its association with dietary habits among female students of Mid‐West University in Surkhet, Nepal. The findings demonstrate that dysmenorrhea is highly prevalent in this population, affecting 88.4% of respondents. This burden is consistent with global reports indicating that dysmenorrhea is one of the most common gynecological conditions among adolescents and young women. A large international meta‐analysis found that ~71% of women worldwide experience dysmenorrhea, with the highest rates observed among university‐aged populations [16]. The severity of pain reported in this study is also noteworthy, as almost half of affected students experienced severe symptoms, aligning with international studies showing that 40%–50% of young women report moderate to severe menstrual pain [4, 28]. Given such high levels of discomfort, the findings highlight dysmenorrhea as a significant but often under‐recognized public health issue that can interfere with students’ cognitive performance, concentration, and daily functioning. Severe menstrual pain is associated with elevated prostaglandin activity, which contributes not only to uterine cramping but also to systemic symptoms such as fatigue and mood changes that may hinder academic engagement [7]. Evidence from university‐based studies further shows that dysmenorrhea is linked to reduced classroom participation, impaired productivity, and diminished health‐related quality of life, suggesting broader functional impacts even when absenteeism is not formally assessed [29, 30]. These mechanisms and observed impacts reinforce the likelihood that students in the present study may also face similar academic and psychosocial challenges despite these outcomes not being measured directly.

The study also found that family history of dysmenorrhea was associated with pain severity, consistent with evidence indicating that hereditary and genetic factors play an important role in menstrual pain. Studies have shown that daughters of women with severe menstrual pain are more likely to experience dysmenorrhea themselves, possibly due to shared physiological pathways involving prostaglandin activity or inherited variations in pain sensitivity [3, 9]. Prostaglandins, particularly prostaglandin F2α, contribute to uterine hypercontractility and reduced uterine blood flow, leading to ischemic pain, and genetic variation may influence the level of prostaglandin production and individual pain thresholds [7]. This pattern in the present study, therefore, reflects both shared biological mechanisms and the intergenerational transmission of pain‐related inflammatory responses.

Research has also reported substantially higher odds of dysmenorrhea among individuals with affected first‐degree relatives, and recent genetic investigations have identified specific loci associated with menstrual pain intensity [31]. In addition, shared familial environments and learned pain‐related behaviors may also influence how pain is perceived and managed [32]. Together, this evidence suggests that family history is a meaningful indicator of underlying biological susceptibility, underscoring the need for early identification and timely management among adolescents and young women.

In contrast, none of the socio‐demographic variables—age, religion, ethnicity, marital status—showed a significant association with pain severity in the current study. This finding accords with other research in which demographic characteristics generally have weak or no association with dysmenorrhea [33, 34]. However, family type showed a significant association in the present study. Given that respondents lived in nuclear, joint, or extended households, this pattern may reflect differences in psychosocial support, family interactions, daily routines, or exposure to stress across household structures. Evidence suggests that stress, emotional climate, and family support can influence how menstrual pain is perceived. For example, one prospective study found that women with higher perceived stress had more than double the risk of dysmenorrhea in the following menstrual cycle, indicating that psychological factors can amplify pain experience [32]. Another cross‐sectional study among adolescents reported that greater stress and lower self‐esteem were associated with more frequent or severe menstrual symptoms [35]. Therefore, the association between family type and pain severity in this study is likely driven by the broader psychosocial environment within different household settings rather than biological differences, highlighting the importance of considering emotional well‐being and social context when addressing menstrual health.

A major aim of this study was to explore whether dietary habits, specific food consumption, or nutritional status are associated with the severity of dysmenorrhea; however, no statistically significant associations were observed. This finding is consistent with previous studies suggesting that, although biologically plausible, the relationship between diet and menstrual pain remains inconsistent. Some studies report protective effects of diets rich in fruits, vegetables, fish, and micronutrients, likely due to anti‐inflammatory and hormonal mechanisms [10, 36], whereas others have found no clear association between dietary patterns and pain severity [11, 37]. The lack of association in this study may be explained by relatively homogeneous dietary practices among university students, as well as the use of self‐reported dietary measures, which may not accurately reflect long‐term nutritional status.

Given that dysmenorrhea is a multifactorial condition influenced by hormonal activity, prostaglandin production, genetic predisposition, and psychosocial factors, the contribution of diet alone may be limited. Similarly, BMI was not associated with pain severity, consistent with evidence showing a weak relationship between body weight and dysmenorrhea [38]. Although extreme BMI levels have been suggested as potential risk factors [9, 39], the predominance of normal BMI in this population may have reduced observable differences.

Dietary practices during menstruation showed that some students avoided specific foods—mostly spicy or sour items—while a minority consumed special foods like soups or chocolate. These behaviors likely reflect a mix of cultural menstrual norms, personal comfort strategies and symptomatic responses (e.g., appetite loss, nausea, bloating), rather than being strictly evidence‐based. Qualitative research among women in rural areas has documented widespread food taboos during menstruation: for example, avoidance of spicy foods, sour fruits, meat, eggs or “hot” foods due to beliefs that such items may worsen bleeding or cause cramps [40, 41]. Similarly, a recent descriptive study found that many menstruating women adjust their diet during periods—consuming more soothing beverages or supplements, and altering meal frequency—when experiencing menstrual symptoms [42]. These findings suggest that food avoidance and selective eating during menstruation are common coping strategies across different cultural and socioeconomic contexts, likely reflecting women's attempts to mitigate discomfort and manage menstrual symptoms.

Most students in the sample used at least one method to manage menstrual pain, but the majority favored home‐based remedies — such as rest, heat therapy, and warm beverages — while only a minority (19.7%) sought medical consultation. This behavior matches patterns observed in other student‐based studies: in a tertiary‐care university setting, many undergraduates reported self‐medication and non‐pharmacological practices, with very few visiting formal health services [43, 44]. The frequent use of over‐the‐counter analgesics such as mefenamic acid is unsurprising, given its widespread recommendation for menstrual pain relief and ready availability in many settings. Even so, reliance on unsupervised medication use—often without professional evaluation—underscores the need for improved menstrual health education and safer pharmacological guidance. Educational programs should emphasize when consulting a healthcare provider is advisable and promote awareness of proper dosing, potential side effects, and underlying causes of severe dysmenorrhea beyond primary menstrual cramps.

The study was completed within a short timeframe and was limited to female bachelor‐level students of a single university, which restricts the generalizability of the findings. Additionally, the use of convenience sampling may introduce selection bias, and self‐reported responses may be subject to recall bias or social desirability bias.

## 5 Conclusion

This study demonstrates a high prevalence of dysmenorrhea among female students at Mid‐West University, with a substantial proportion experiencing moderate to severe pain. Family history was significantly associated with pain severity, highlighting the role of biological and hereditary factors, while dietary habits and BMI showed no significant associations. Despite the high burden, most students relied on self‐care practices and did not seek medical consultation. These findings emphasize the need for improved menstrual health education and access to appropriate pain management among university students.

Future studies should adopt longitudinal or interventional designs to better understand causal relationships between lifestyle factors and dysmenorrhea. Incorporating objective measures such as dietary assessments and biological markers may help clarify underlying mechanisms. Additionally, research exploring healthcare‐seeking behaviors and barriers to medical consultation among students would provide valuable insights for targeted interventions.

## Author Contributions

**Rashmi Pant:** conceptualization, investigation, methodology, supervision, funding acquisition, visualization, project administration, writing – original draft. **Mamta Verma:** methodology, software, data curation, formal analysis, visualization, resources, writing – original draft, writing – review and editing. **Rima Mishra:** methodology, software, data curation, investigation, validation, formal analysis, writing – review and editing. **Ganesh Bhandari:** conceptualization, methodology, data curation, investigation, validation, formal analysis, supervision, writing – original draft, writing – review and editing.

## Funding

The authors have nothing to report.

## Conflicts of Interest

All the authors declare no conflicts of interest.

## Transparency Statement

The lead author Ganesh Bhandari affirms that this manuscript is an honest, accurate, and transparent account of the study being reported; that no important aspects of the study have been omitted; and that any discrepancies from the study as planned (and, if relevant, registered) have been explained.

## Data Availability

The data supporting the findings of this study are available from the corresponding author upon reasonable request. Due to ethical restrictions related to protecting participant confidentiality (as participants were assured that individual responses would not be shared publicly), the dataset is not publicly archived. The questionnaire used for data collection is provided as Supporting File 1.
